# The impact of the OPRM1, OPRK1, DCC genes polymorphisms on the motivation for non-suicidal self-injury in young adults: a pilot study

**DOI:** 10.1192/j.eurpsy.2023.2353

**Published:** 2023-07-19

**Authors:** A. A. Kibitov, G. E. Mazo

**Affiliations:** 1 Translational Psychiatry Department, Bekhterev National Medical Research Center for Psychiatry and Neurology, Saint Petersburg, Russian Federation

## Abstract

**Introduction:**

Non-suicidal self-injury (NSSI) – the deliberate, self-directed damage of body tissue without suicidal intent and for purposes not socially or culturally sanctioned – is a highly prevalent phenomenon in adolescents and young adults. Motivation for the NSSI is known to be heterogenous in different patients. However, biological and especially genetic markers associated with different motivation for the NSSI have not been studied to date. One of the possible candidates are mu- (*OPRM1*) and kappa-opioid receptor (*OPRK1*) genes, since opioid system is known to be involved in the NSSI. Another perspective candidate is the *DCC* gene, encoding the netrin 1 receptor, which plays vital part in the formation of the prefrontal cortex.

**Objectives:**

We conducted a pilot cross-sectional study to test the impact of the rs1779971 *OPRM1,* rs6473797 *OPRK1* and rs8084280 *DCC* gene polymorphisms on the characteristics and motivations for the NSSI in young adults.

**Methods:**

28 patients of European ancestry with NSSI (89,3%; n=25) women, median age (Q1-Q3) – 23 (21,25-25) years) were included in the pilot sample. The majority of the sample had a diagnosis of bipolar disorder (78,6%; n=20). Characteristics and motivation for the NSSI were measured by the Inventory of Statements about Self-Injury (ISAS). The Childhood Trauma Questionnaire (CTQ) was used to control for adverse childhood experiences, a potent environmental factor, associated with NSSI. Genotyping was performed using RT-PCR. The genetic effect was evaluated using the dominant model. For statistical analysis multiple linear regression with the presence of minor alleles, different types of childhood trauma, diagnosis, age and sex as factors and ISAS scores as dependent variables were used.

**Results:**

Multiple linear regression showed that minor C allele of rs6473796 *OPRK1* gene polymorphism was associated with an increase of the “Affect regulation” (B=2,23; CI95% [0,39–4,06]; p=0,022), “Anti-dissociation” (B=3,31; CI95% [0,18–6,44]; p=0,039) subscales of ISAS scores. Moreover, the minor T allele of the rs8082480 *DCC* gene polymorphism was associated with a decrease of the “Affect regulation” subscale score (B=-1,74; CI95% [-3,30 – -0,18]; p=0,032).

**Conclusions:**

To our knowledge, this is the first study on the genetic markers of motivations for NSSI. Our pilot results showed that, controlling for diagnosis, age, sex and childhood trauma, *OPRK1* and *DCC* gene polymorphisms may be associated with the heterogeneity of motivations for NSSI. However, these results require confirmation on the larger samples.

**Disclosure of Interest:**

None Declared

